# The Prevalence of *Angiostrongylus cantonensis/mackerrasae* Complex in Molluscs from the Sydney Region

**DOI:** 10.1371/journal.pone.0128128

**Published:** 2015-05-22

**Authors:** Douglas Chan, Joel Barratt, Tamalee Roberts, Rogan Lee, Michael Shea, Deborah Marriott, John Harkness, Richard Malik, Malcolm Jones, Mahdis Aghazadeh, John Ellis, Damien Stark

**Affiliations:** 1 Department of Microbiology, SydPath, St. Vincent’s Hospital, Victoria St, Darlinghurst, NSW, Australia; 2 i3 Institute, University of Technology, Sydney, Ultimo, NSW, Australia; 3 School of Medical and Molecular Sciences, University of Technology, Sydney, Ultimo, NSW, Australia; 4 Centre for Infectious Diseases and Microbiology Laboratory Services, ICPMR, Westmead Hospital, Westmead, NSW, Australia; 5 Malacology Department, Australian Museum, Sydney, NSW, Australia; 6 Centre for Veterinary Education, University of Sydney, Camperdown, NSW, Australia; 7 School of Veterinary Science, The University of Queensland, Gatton, Queensland, Australia; Royal Tropical Institute, NETHERLANDS

## Abstract

*Angiostrongylus cantonensis* and *Angiostrongylus mackerrasae* are metastrongyloid nematodes that infect various rat species. Terrestrial and aquatic molluscs are intermediate hosts of these worms while humans and dogs are accidental hosts. *Angiostrongylus cantonensis* is the major cause of angiostrongyliasis, a disease characterised by eosinophilic meningitis. Although both *A*. *cantonensis* and *A*. *mackerrasae* are found in Australia, *A*. *cantonensis* appears to account for most infections in humans and animals. Due to the occurrence of several severe clinical cases in Sydney and Brisbane, the need for epidemiological studies on angiostrongyliasis in this region has become apparent. In the present study, a conventional PCR and a TaqMan assay were compared for their ability to amplify *Angiostrongylus* DNA from DNA extracted from molluscs. The TaqMan assay was more sensitive, capable of detecting the DNA equivalent to one hundredth of a nematode larva. Therefore, the TaqMan assay was used to screen molluscs (n=500) of 14 species collected from the Sydney region. *Angiostrongylus* DNA was detected in 2 of the 14 mollusc species; *Cornu aspersum* [14/312 (4.5%)], and *Bradybaenia similaris* [1/10 (10%)], which are non-native terrestrial snails commonly found in urban habitats. The prevalence of *Angiostrongylus* spp. was 3.0% ± 0.8% (CI 95%). Additionally, experimentally infected *Austropeplea lessoni* snails shed *A*. *cantonensis* larvae in their mucus, implicating mucus as a source of infection. This is the first Australian study to survey molluscs using real-time PCR and confirms that the garden snail, *C*. *aspersum*, is a common intermediate host for *Angiostrongylus* spp. in Sydney.

## Introduction

*Angiostrongylus cantonensis* and *Angiostrongylus mackerrasae* are metastrongyloid nematodes that infect various rat species. Colloquially, they are referred to as rat lungworms, although they actually reside in the pulmonary arteries, but lay eggs that embolise to the pulmonary parenchyma, where they hatch giving rise to L_1_ larvae. *Angiostrongylus cantonensis* is endemic to Southeast Asia, the Pacific Islands, Hawaii, the Americas, the Caribbean and eastern Australia, though new epidemiological data and recent clinical cases suggest a possible expansion of the parasites geographical range [1]. *Angiostrongylus mackerrasae* is a close relative of *A*. *cantonensis* [2], and is extremely difficult to differentiate from *A*. *cantonensis* morphologically [3]. While *A*. *cantonensis* and *A*. *mackerrasae* are both found in Australia, *A*. *cantonensis* appears to account for most infections in humans, domestic animals and wildlife. *Angiostrongylus cantonensis* is considered to be the main cause of eosinophilic meningitis worldwide [4].

Several rat species are recognised as definitive hosts of *A*. *cantonensis*, while terrestrial and freshwater molluscs are intermediate hosts [1,5,6]. In Australia, two exotic rat species; *Rattus rattus* and *Rattus norvegicus* are the preferred definitive hosts for *A*. *cantonensis*, which appears to have a lower propensity to infect native rat species such as *Rattus fuscipes*. Inversely, *Rattus fuscipes* is thought to be an important definitive host for *A*. *mackerrasae* [7]. Paratenic hosts (i.e. hosts in which the parasite remains quiescent in tissues) for *Angiostrongylus* spp. include crabs, frogs, reptiles and fish [8]. Several large vertebrates (i.e. mammals and birds, including humans) are considered accidental or dead-end hosts of *A*. *cantonensis*. In these hosts, the worms travel to the peripheral and/or central nervous system to mature though are unable to complete their lifecycle [8]. Accidental hosts may become infected following the intentional or unintentional ingestion of raw or undercooked molluscs, or a paratenic host [9–11], harbouring the infective L_3_ larval form of the parasite. It has been suggested that infective *A*. *cantonensis* larvae are also shed in mollusc mucus. This may be a source of infection for those who regularly eat uncooked plant material that may be contaminated with mucus from infected slugs and snails [12,13].

The clinical manifestations associated with angiostrongyliasis include headache, neck stiffness, paraesthesia, vomiting and nausea [4]. If untreated, the disease progresses to encephalitis, meningoencephalitis or death [14]. A diagnosis of *Angiostrongylus* eosinophilic meningitis is often overlooked as the disease is relatively rare and the clinical signs are non-specific.

A conventional PCR assay was developed by the Centers for Disease Control and Prevention (CDC) to survey local mollusc populations in the Hawaiian Islands, in response to a local outbreak of *A*. *cantonensis* infection [15]. This assay detects approximately one *Angiostrongylus* larva per milligram of mollusc tissue, although cross reaction with DNA from another strongylid worm was reported [15]. Three years later, a TaqMan real-time PCR assay was developed by the same investigators, for the detection of *Angiostrongylus* DNA [16]. This assay has been evaluated on human CSF samples in severe angiostrongyliasis cases in Hawaii, though the sample size was limited [17]. While there is limited information on its applicability in a clinical setting, the TaqMan assay has been employed in multiple studies to determine the prevalence of *A*. *cantonensis* in molluscs collected in field studies [10,18–20].

Severe clinical cases of angiostrongyliasis have been reported in Australia, mostly in the Sydney and Brisbane areas. The first case was reported in 1971 near Brisbane [21], and 34 severe cases have been reported in Australia since then [6,22–24]. Most recently three severe cases (one lethal) were reported in Sydney [23,24], resulting in increased local concern in this rare disease. These cases highlight the need to generate new epidemiological data for angiostrongyliasis in this region. To address this issue and facilitate an assessment of the potential risk of angiostrongyliasis in this region, this study compared the conventional PCR and TaqMan assay developed by Qvarnstrom *et al*. [15,16] for their ability to amplify *Angiostrongylus* DNA in DNA extracted from mollusc tissues collected throughout NSW but focussed on the Sydney area.

## Materials and Methods

### Ethics statement

Ethics approval was obtained from the St Vincent’s Hospital research office, (HREC reference number: LNR/13/SVH/361). Samples collected from horticultural facilities and private properties were done with the permission from property owners involved. This research did not involve the use of endangered or protected mollusc species.

### Mollusc collection

All molluscs were collected between the months of February and August, 2014 from different locations throughout New South Wales, Australia. Collection points mostly included locations in the greater Sydney metropolitan area (Table 1). Habitats from which molluscs were collected included local parks, gardens, nurseries, ponds, train stations, forests, private properties and mail boxes. Live molluscs were stored in plastic collection containers for transport to the laboratory where they were immediately killed by freezing for at least 3 hours. Specimens were stored at -18°C until required for DNA extraction. Molluscs collected in the field were photographed using a digital camera from different angles for later identification by one of the authors (M. Shea). In total, 500 molluscs were collected, identified and tested for the presence of *Angiostrongylus* DNA. A total of 14 mollusc species were identified, including eight terrestrial species and six aquatic species of mollusc (Table 2).

**Table 1:** Suburbs where molluscs were collected in New South Wales.

Suburb	Postcode	Geographic Coordinates
Alexandria	2015	-33.907, 151.192
Annandale	2038	-33.881, 151.171
Balmain	2041	-33.85, 151.184
Bondi^a^	2026	-33.89, 151.272
Burwood^a^	2134	-33.855, 151.184
Camperdown	2050	-33.886, 151.175
Campsie	2194	-33.914, 151.103
Caringbah	2229	-34.044, 151.119
Carlton	2218	-33.971, 151.120
Carss Park	2221	-33.987, 151.104
Casula	2170	-33.931, 150.914
Drummoyne	2047	-33.852, 151.154
Goulburn	2580	-34.755, 149.718
Haberfield	2045	-33.880, 151.139
Hurstville^a^	2220	-33.967, 151.101
Kingsgrove	2208	-33.942, 151.101
Kogarah	2217	-33.972, 151.136
Lane Cove	2066	-33.818, 151.162
Neutral Bay	2089	-33.837, 151.219
Newtown	2042	-33.900, 151.177
North Sydney^a^	2060	-33.839, 151.206
Oakville	2765	-33.647, 150.841
Paddington	2021	-33.885, 151.226
Padstow	2211	-33.956, 151.032
Parklea	2768	-33.729, 150.931
Petersham	2049	-33.895, 151.152
Redfern^a^	2016	-33.893, 151.207
Richmond	2753	-33.598, 150.753
Riverwood	2210	-33.964, 151.053
Rozelle	2039	-33.859, 151.174
St Ives	2075	-33.725, 151.169
Surry Hills	2010	-33.883, 151.216
Sydney Botanical Gardens	2000	-33.870, 151.210
Sylvania	2224	-34.012, 151.103
The Entrance	2261	-33.359, 151.456
Turramurra	2074	-33.707, 151.127
University of Sydney	2006	-33.888, 151.187
Warwick Farm	2170	-33.931, 150.914
Wentworth Falls	2782	-33.729, 150.379
Willoughby^a^	2068	-33.800, 151.212
Windsor	2756	-33.317, 151.026
Zetland	2017	-33.904, 151.207

^a^Suburbs where *Angiostrongylus cantonensis/mackerrasae* was detected.

**Table 2:** Molluscan species collected within the study and the number of samples of each species.

Species Name	Family	Habitat	Number
*Cornu aspersum* ^*a*^	*Helicidae*	Terrestrial	312
*Lehmannia nyctelia*	*Limacidae*	Terrestrial	76
*Physa acuta*	*Physidae*	Aquatic	24
*Limacus flavus*	*Limacidae*	Terrestrial	20
*Limax maximus*	*Limacidae*	Terrestrial	17
*Pseudosuccinea columella*	*Lymnaeidae*	Aquatic	13
*Bradybaena similaris* ^a^	*Bradybaenidae*	Terrestrial	10
*Deroceras invadens*	*Agriolimacidae*	Terrestrial	9
*Phallomedusa solida*	*Littorinidae*	Aquatic	7
*Nerita atramentosa*	*Neritidae*	Aquatic	5
*Bembicium auratum*	*Littorinidae*	Aquatic	3
*Austropeplea lessoni*	*Lymnaediae*	Aquatic	2
*Milax gagates*	*Milacidae*	Terrestrial	1
*Austrorhytida capillacea*	*Rhytididae*	Terrestrial	1

^a^ Species in which *Angiostrongylus cantonensis/mackerrasae* DNA was detected.

### Preparation of infected mollusc controls

To prepare positive controls for this study, an Australian native freshwater mollusc, *Austropeplea lessoni*, was infected in a controlled environment (an indoor freshwater aquarium), known to be free of *Angiostrongylus* spp. Briefly, L_1_ larvae of *A*. *cantonensis* were obtained from the faeces of rats experimentally infected with *A*. *cantonensis*, using the Baermann technique [25]. Positive control molluscs were generated by placing the molluscs into a deep petri dish and allowing them to naturally take up L_1_ larvae. Several *A*. *lessoni* snails were kept uninfected for use as negative controls. Positive and negative *A*. *lessoni* were housed in different tanks, with a different water supply, to prevent cross infection between the positive and negative groups.

### Selecting mollusc tissues for DNA extraction

**Fig 1:**
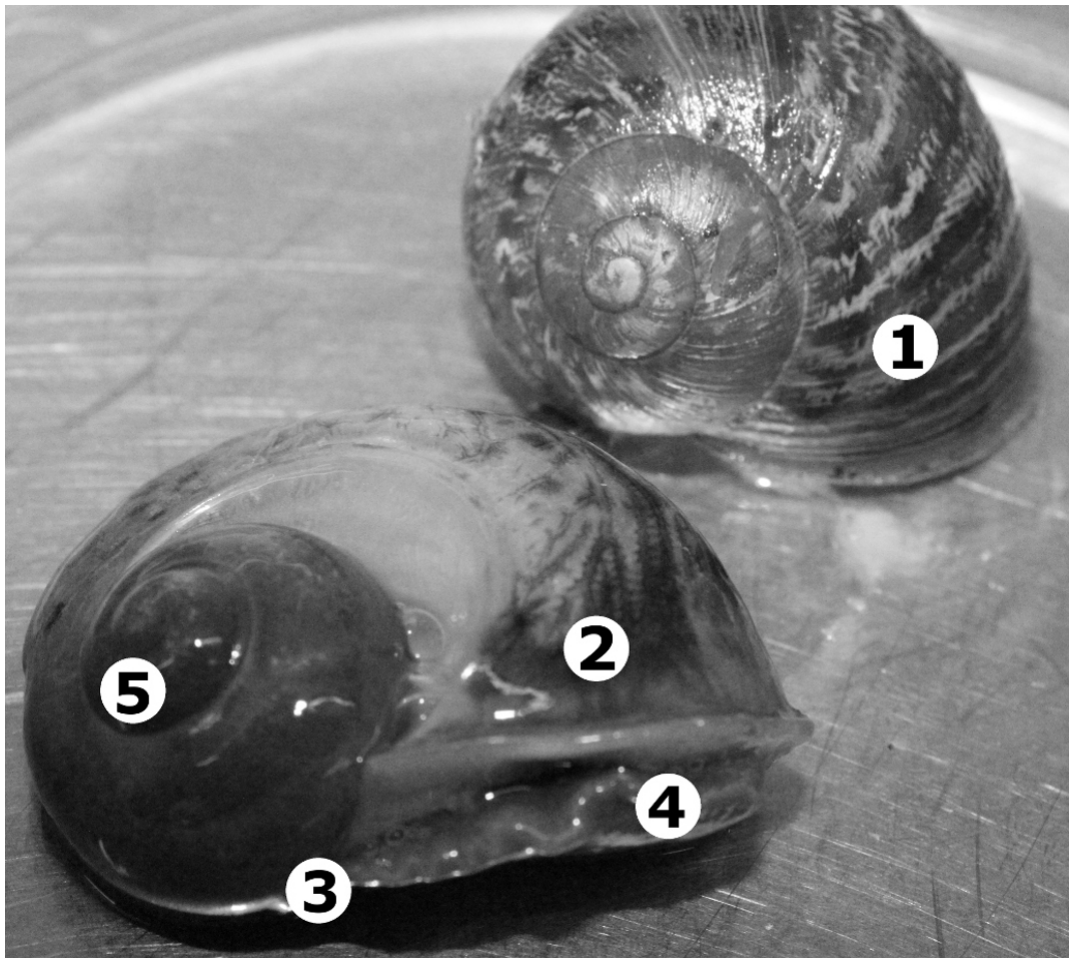
Basic anatomy of snails, using the common garden snail (*Cornu aspersum*) as an example. Numbers in the figure show the basic anatomical features of the mollusc. (1) Mollusc shell, (2) Mantle, (3) Foot/Tail, (4) Head, (5) Digestive tract.

To select an appropriate mollusc tissue from which to extract DNA, whole snails were roughly divided into five major anatomical regions, which included the shell, mantle, foot/tail, head, and the digestive tract (Fig 1). These anatomical regions (excluding the shell) were examined microscopically using experimentally infected *A*. *lessoni*. The regions with the highest parasite loads were selected, and dissected from snails collected in the field for DNA extraction. This was particularly necessary for larger snails for which extraction of DNA from the entire specimen was not feasible. Three experimentally infected *A*. *lessoni* were examined microscopically several days post-infection. To quantify parasite loads in different anatomical regions, shells were removed first by cracking and removing individual pieces using forceps. The molluscs were then placed in a customized glass clamp for examination of tissues using stereo-microscopy and the number of *A*. *cantonensis* larvae present in each anatomical region was counted manually. Mollusc tissue from the foot and mantle of the snail were chosen for extraction of genomic DNA.

### Extraction of genomic DNA

Using a sterile disposable scalpel, frozen tissue was cut from the posterior portion of the foot and mantle of infected molluscs. Tissue samples were then crushed and sliced using the scalpel to facilitate subsequent digestion. Approximately 50 mg of mollusc tissue was placed in a 1.5 mL Eppendorf tube and DNA was extracted using an EZ1 DNA tissue kit and the EZ1 Biorobot (Qiagen) following the manufacturer’s protocol. Briefly, molluscan tissue was treated with 10 μL of proteinase K solution and 190 μL of tissue digestion buffer G2 (provided in the EZ1 DNA tissue kit) and vortexed for approximately 15 seconds. Samples were then digested for approximately 24 hours at 56°C using a dry block heater (Ratek). The tubes were vortexed at regular intervals throughout this period until complete digestion of the tissue was achieved. Immediately before extraction, tissue lysates were vortexed for approximately 15 seconds and then centrifuged at 500 g for 20 seconds to pellet any suspended material. The supernatant was retained and DNA was extracted from this solution using the EZ1 Biorobot. Extracted DNA was stored at -18°C until required for PCR analysis.

### Conventional PCR

A conventional PCR assay was adapted from an assay described by Qvarnstrom *et al* [15], and validated in-house. To begin, DNA extracted from the foot and mantle of experimentally infected *A*. *lessoni* were used. Primers AngioF1 and AngioR1 (S1 Table), originally described by Qvarnstrom *et al* [15] were purchased from Sigma Aldrich. Reactions were prepared to contain 0.4 μM of AngioF1, 0.4 μM of AngioR1, 2 μL of DNA template, a single Illustra PureTaq Ready-To-Go PCR bead (Amersham Pharmacia Biotech), and PCR quality water to a total volume of 25 μL.

Reaction mixtures were gently mixed before amplifications were carried out on a Multi Gene II Thermocycler. Temperature cycling conditions were as follows: 95°C for 5 minutes, followed by 30 cycles of 95°C for 15 seconds, 60°C for 30 seconds and an extension step of 72°C for 60 seconds. This was followed by a final extension step of 72°C for 10 minutes. The PCR products were held in the thermocycler at 4°C until required for electrophoresis. Each PCR run was accompanied by negative controls consisting of DNA extracted from an uninfected mollusc and another negative control consisting of 2 μl of H_2_O as template instead of DNA.

### Gel Electrophoresis

Amplified PCR products from conventional PCR reactions were analysed by gel electrophoresis on pre-cast E-gel EX 2% agarose plates (Life Technologies). E-gel plates contained 11 wells and SYBR Gold dye. Amplified DNA was prepared for gel loading by adding 15 μL of PCR water to 5 μL of sample before loading into wells. Additionally, an E-Gel 1Kb Plus DNA ladder (Invitrogen) was loaded into the first well followed by samples. The PCR products were electrophoresed for 10 minutes in an E-Gel iBase (Life Technologies) before visualisation of bands under UV light.

### Sequencing the complete *A*. *mackaressae* and *A*. *cantonensis* 18S genes

PCR products were generated from the 18S rRNA gene of *A*. *mackaressae* and *A*. *cantonensis* using the conventional PCR protocol described previously. PCR products were diluted in a 5:1 ratio with PCR water and purified using a QIAquick PCR purification kit (Qiagen) according to the manufacturer’s instructions with the following alteration; products were eluted using 30 μL volumes instead of 50 μL volumes during the final step in the protocol to obtain an increased DNA concentration. Products were run on 2% agarose gels next to a E-Gel Low Range Quantitative DNA ladder to approximate the DNA concentration in the purified PCR products. The purified PCR products were prepared in 7 μL volumes, containing 2 μL of PCR primer, 3uL of DNA (~60ng), and 2 μL of PCR water. All PCR products were sequenced once using each of the sequencing primers NEM2F, NEM3F, NEM3R and NEM3F (S1 Table) described by Qvarnstrom *et al*. [15]. Each of these solutions was sent to the Australian Genome Research Facility for capillary sequencing.

The chromatogram files returned for each PCR product were analysed using the Sequence Scanner v.2 software suite (Life Technologies) to trim and remove the low quality ends of sequences. The quality controlled sequences were assembled, edited and aligned using Sequence Scanner v.2. The identity of each sequence was confirmed using online BLAST searches against the NCBI nucleotide database, to confirm homology to *Angiostrongylus* spp. 18S sequences.

### TaqMan Real-Time PCR

The real-time PCR used in this study was based on the TaqMan assay previously described by Qvarnstrom *et al*. [16]. The PCR primers and the TaqMan probe were purchased from Sigma-Aldrich. Reactions were prepared in a total reaction volume of 20 μL in smart cycler tubes (Cepheid) to contain 0.2 μM of forward primer AcanITSF1, 0.2 μM of reverse primer AcanITSR1, 0.05 μM of probe AcanITSP1, 2 μL of DNA template and 10 μL of SensiFast Probe No-ROX mix (Bioline). All reactions were prepared on a frozen block and upon completion of the reaction setup, were centrifuged for 10 seconds to move reaction contents to the bottom of the smart cycler tubes. All real-time PCR reactions were subjected to the following temperature cycling conditions: 40 cycles of 95°C for 15 seconds followed by 60°C for 45 seconds. Each PCR run was accompanied by negative controls consisting of DNA extracted from an uninfected mollusc and another that used 2 μL of H_2_O as template instead of DNA. All reactions were carried out on a Smart Cycler II (Cepheid).

### Limits of detection and calculation of real-time PCR efficiency

To determine the limit of detection for all PCR assays, *A*. *lessoni* snails infected with L_3_ *A*. *cantonensis* larvae were used. The *A*. *lessoni* shells were removed as previously described and the digestive organs were discarded. The remaining tissue was subjected to pepsin/HCl digestion to liberate L_3_ larvae for microscopic quantification [15]. Briefly, molluscan tissue was treated with a 2 mL solution containing 0.1% pepsin and 1% HCl solution in a 2 mL Eppendorf tube. Tubes containing the tissue and pepsin/HCL solution were placed on a heat block at 37°C with vortexing at 30 minute intervals over at least 4 hours or until complete digestion of tissues was achieved. Solutions containing liberated L_3_ larvae were then suspended by gently shaking the tube. Then, 100 μL of the suspension was pipetted onto a microscope slide. The L_3_ *A*. *cantonensis* larvae were then counted manually. This process was repeated in triplicate to accurately quantify the parasite concentration within these solutions. Based on these counts, a volume of parasite suspension containing approximately 100 L_3_ *A*. *cantonensis* larvae was removed from the suspension. These ~100 larvae were then placed into a separate tube and centrifuged at approximately 15,800 g for 5 minutes. The supernatant was carefully removed, leaving only the pellet containing the parasites. Approximately, 50 mg of mollusc tissue known to be negative for *Angiostrongylus* spp. was added to these 100 L_3_ larvae. DNA was then extracted from the L_3_ larvae and mollusc tissue using the DNA extraction protocol described previously. Extracted DNA was then serially diluted with PCR water into separate Eppendorf tubes to prepare DNA solutions containing DNA equivalent to 100, 10, 1, 0.1, 0.01 L_3_ larvae. Tubes were then stored at -18°C until required for downstream molecular analyses. Each of these serial dilutions was used as template for the conventional and real-time PCR assays in order to confirm the limit of detection (i.e. sensitivity) of both assays.

To calculate the RT-PCR efficiency and determine the limit of detection for the assay, DNA extracted from 0.01, 0.1, 1, 10 and 100 L_3_ *A*. *contonensis* larvae was amplified using the TaqMan assay and a standard curve was plotted from the cycle threshold (Ct) values obtained. The TaqMan amplification efficiency was calculated using this information. Briefly, the efficiency of the TaqMan PCR reaction was calculated using the following equation: Efficiency = -1+10^(-1/slope)^.

### Inhibition controls

To control for potential PCR inhibitors in genomic DNA extracts, universal eukaryotic primers, F-573 and R-1200 (S1 Table) [26] were used to amplify a fragment of DNA from the molluscan 18S rRNA gene of each sample. The PCR protocol used was identical to that described for the conventional PCR, although primers F-573 and R-1200 were used instead of AngioF1 and AngioR1. This was performed to rule out false negatives resulting from PCR inhibition. As an additional measure to rule out inhibition in the Real-Time PCR assay, 10 μL of each DNA sample was spiked with 2 μL of *A*. *cantonensis* DNA and then analysed on the Smart Cycler II (Cepheid), using the Real-Time PCR protocol described. Samples suspected of containing inhibitors were diluted with PCR water and then retested.

### Specificity controls

To assess the specificity of each PCR assay, the assays were applied to DNA extracted from several helminth species to determine if a PCR product or positive Ct result was produced. The assays were tested on DNA extracted from: *Angiostrongylus mackerrasae*, *Angiostrongylus cantonensis* (positive control), *Strongyloides stercoralis*, *Trichuris trichiura*, *Taenia* spp., *Ascaris lumbricoides*, *Ascaris suum*, *Dipylidium caninum* and *Diphyllobothrium* spp.

### Prevalence and statistical significance

The prevalence of *Angiostrongylus* spp. was calculated from the number of molluscs collected and the number of *Angiostrongylus cantonensis*/*mackerrasae* positive samples determined by the TaqMan assay using a 95% confidence interval. This calculation was used to determine the prevalence in the entire sample and in each mollusc individually.

### Cloning of *A*. *cantonensis* and *A*. *mackerrasae* ITS1 DNA

PCR products were generated from the ITS-1 of *A*. *cantonensis* and *A*. *mackerrasae* using a PCR assay based on that of Qvarnstrom *et al*. [16]. PCR mixtures were prepared to contain 0.4 μM of forward primer (AngioF1674) and 0.4 μM of reverse primer (58SR4), 2 μl of DNA template and a single Illustra PureTaq Ready-To-Go PCR bead, in a total volume of 25 μL. This reaction was used to amplify an 870 bp fragment of the ITS-1 DNA region from both *Angiostrongylus* species. Initially, PCR products were generated and sequenced as described for the conventional PCR. However, the chromatograms generated were difficult to interpret, presumably due to the significant variability between individual copies of the ITS-1 DNA region. Cloning was therefore carried out so that copies of this region could be sequenced individually. As the 18S genes were found to be virtually identical between *A*. *cantonensis* and *A*. *mackerrasae* (see results) and ITS-1 DNA is more susceptible to mutation than the 18S genes, ITS-1 sequencing was carried out to determine whether significant differences might exist between the ITS-1 regions of these worms which may enable their differentiation.

Amplified ITS-1 PCR products were cloned using a TOPO TA cloning kit (Life Technologies) in accordance with the manufacturer’s instructions. Several colonies containing the ITS1 amplicons of *A*. *mackerrasae* and *A*. *cantonensis* were then picked individually on the selective agar and cultured in separate falcon tubes containing 5 mL of LB broth with 50 μg/mL of Ampicillin. This LB broth was incubated overnight at 37°C before plasmid DNA isolation.

### Plasmid DNA isolation

Saturated *E*. *coli* broth (2 mL) containing the transformants was placed in individual Eppendorf tubes and centrifuged at 11,000g for 30 seconds to pellet the cells. The supernatant was carefully decanted. Isolation of plasmid from the resulting cell pellets was achieved using the ISOLATE II Plasmid Mini kit (Bioline) with the following modification: the optional wash buffer step was performed for improved DNA sequencing reads.

### Sequencing of plasmid DNA

Sequencing of the ITS-1 PCRs were carried out as previously described, using the plasmid as template. This was carried out as a control to confirm successful cloning. The concentration of plasmid solutions was confirmed using a NanoDrop ND-1000 UV spectrophotometer. The plasmid solutions were diluted in accordance with the guidelines provided by the service provider (Macrogen Inc, Korea), where the sequencing of the plasmid inserts was carried out using the universal plasmid primers T3 and T7. Analysis of chromatograms returned by Macrogen Inc. was carried out using Chromas Lite software (http://technelysium.com.au). After viewing the chromatograms in Chromas Lite, the low quality ends of sequences and any residual vector sequence was removed from each sequence. The resulting quality controlled sequences were then assembled manually by alignment using ClustalW. Online BLASTN searches against the NCBI nucleotide database were carried out on each resulting contig, to confirm homology to *Angiostrongylus* spp. ITS-1 sequences. All ITS-1 contigs were compared to each other by ClustalW alignment to determine whether substantial differences existed between the ITS-1 DNA regions of *A*. *mackerrasae* and *A*. *cantonensis*.

### Infectivity of Mollusc Mucus

Mollusc mucus was collected from both infected and uninfected *Austropeplea lessoni* mollusc controls. Molluscs were placed in a sterile petri dish and stimulated to create mucus by gently prodding the flesh using a sterile wooden applicator sticks. Mucus secretions (~50 μL) were collected in Eppendorf tubes and thinned by the addition of 50 μL of PCR water. Secretions were first examined microscopically for the presence of any nematode species before being subjected to DNA extraction and RT-PCR analysis to confirm the presence or absence of *Angiostrongylus* spp. in mucus.

## Results

### Assay specificity, sensitivity and TaqMan assay efficiency

The conventional PCR ‘which’ amplified a 1,134bp fragment of the 18S rRNA gene was the least sensitive of the assays as it was unable to detect fewer than 10 parasites equivalent of extracted DNA in a background of mollusc DNA. The real-time PCR assay was the most sensitive technique, as it was able to detect the DNA equivalent of 0.01 of a parasite (Ct = 35.68). The TaqMan assay was thus 1000 times more sensitive than the conventional PCR protocol. Due to its superior sensitivity, the Real-Time PCR assay was employed to screen all DNA extracts originating from molluscs collected in the field. After plotting the standard curve, the TaqMan real-time PCR efficiency was calculated to be 106.5% and with an R^2^ value of 0.996. Both PCR assays amplified products from *A*. *mackerrasae* as well as *A*. *cantonensis* but did not amplify products for DNA extracted from the other helminths tested.

### Real-Time PCR prevalence of *A*. *cantonensis/mackerrasae* complex

The TaqMan assay detected *A*. *cantonensis/mackerrasae* DNA in 3% (15/500) of mollusc DNA extracts. Of these 15 samples, parasite loads were low in all 15 of these samples. Two samples; one *B*. *similaris* and one *C*. *aspersum*, had parasite loads of approximately 1 parasite per 50 mg of tissue while the other positives (13) had parasite loads of approximately 0.01–0.1 parasite per 50mg of mollusc tissue. The prevalence of *Angiostrongylus* spp. was calculated at 3% ± a standard error of 0.76% (95% confidence interval [Z-score] of 1.96). *Angiostrongylus* spp. was detected in two species of mollusc, *C*. *aspersum* [14/312; 4.50% ± 2.30% (CI 95%)] and *B*. *similaris* [1/10; (10%)]. The locations where this complex was detected can be seen in Table 2.

### Sequencing of cloned ITS-1 PCR products

The ITS1 region of *A*. *cantonensis* and *A*. *mackerrasae* were successfully amplified using the conventional PCR assay. A product of approximately 830bp from *A*. *cantonensis* and *A*. *mackerrasae* DNA was successfully cloned into the TOPO Vector based on PCR results obtained using purified plasmid extracted from transformants as template. Seven *E*. *coli* colonies containing *A*. *mackerrasae* and four colonies containing *A*. *cantonensis* amplicons produced a clean sequence following sequencing in the forward and reverse directions. BLASTN searches against the NCBI database confirmed that the PCR assay had amplified the ITS1 region of *A*. *cantonensis* and *A*. *mackerrasae* (each clone achieved an e-value of 0.0 compared to other *A*. *cantonensis* ITS-1 sequences available in the NCBI database). Pairwise alignment of cloned sequences from both species confirmed that the ITS-1 regions were highly similar (<2%) to each other.

### Sequencing results of the 18S rRNA

All chromatogram files for the 18S rRNA region of both *A*. *cantonensis* and *A*. *mackerrasae* were assembled. A BLASTN search of the sequenced 1067 bp 18S rRNA gene fragment against the NCBI nucleotide database confirmed that the PCR assay had amplified the *A*. *cantonensis* and *A*. *mackerrasae* 18S genes. The sequence generated for *A*. *cantonensis* received top blast hits to an *A*. *cantonensis* 18S gene already available in the database (100% identity, E value = 0.0). Similarly the 18S rRNA contig obtained for *A*. *mackerrasae* was 100% identical to that of *A*. *cantonensis* (E value = 0.0).

### Mollusc mucus as a source of Angiostrongylus spp. infection

Analysis of mollusc mucus from control specimens revealed the presence of *A*. *cantonensis* larvae microscopically and this was quantitated by qPCR. The qPCR was able to detect an average parasite load of approximately 20–40 larvae per ~50 μL sample of mucus. There was no evidence of *A*. *cantonensis* DNA from negative controls/uninfected molluscs.

## Discussion

Angiostrongyliasis is a rare disease in humans caused by the rat lungworm (*A*. *cantonensis*). The occurrence of recent clinical cases of this disease in the Sydney area highlights the need to generate up-to-date data on the range and prevalence of *Angiostrongylus* spp. in the region. To address this need, a PCR survey was carried out using a previously described TaqMan assay [16] to determine the prevalence of *Angiostrongylus* spp. in molluscs collected in the greater Sydney metropolitan area.

Based on the sequencing analysis performed in this study, *A*. *mackerrasae* and *A*. *cantonensis* are remarkably similar genetically. As such, it is not surprising that the TaqMan assay detects DNA from both species. Sequencing analysis of the *A*. *mackerrasae* 18S gene carried out in this study (Genbank accession KP776404), confirmed that these genes are identical for *A*. *mackerrasae* and *A*. *cantonensis* at least for the region sequenced. Furthermore, sequencing of the ITS1 gene clones generated in this study revealed little to no difference (~2% at most) between the ITS1 from these two species (Genbank accessions KP776405 to KP776415). This makes designing of species-specific primers from these loci particularly difficult. Qvarnstrom *et al*. [16] had originally stated that the variability in the ITS1 region makes it possible for the TaqMan assay to differentiate members of the *Angiostrongylus* genus to the species level. We can confirm that this is not the case for *A*. *mackerrasae* and *A*. *cantonensis*. Indeed, based on the similarities observed in this study, it is possible that these worms belong to the same species, possibly as different subspecies. However, the taxonomic position of these worms will become clearer as more sequence datum become available.

While *A*. *mackerrasae* and *A*. *cantonensis* are both endemic to Australia (Prociv and Carlisle, 2001), the clinical importance of *A*. *mackerrasae* remains unclear. A weakness of this study is that *A*. *mackerrasae* is believed to be less important clinically and the results do not indicate which species was detected in the molluscs sampled. However, given the current difficulties associated with differentiation of the two species (genetically and morphologically) it is possible that some clinical cases that have occurred in Australia might be attributable to *A*. *mackerrasae*. The clinical importance of *A*. *mackerrasae* will become clearer as more molecular data become available for these species, such that their differentiation by molecular testing is possible. Based on the results of a previous study, examination of the *A*. *mackerrasae* COI gene may be useful. Based on this report [27], which included phylogenetic analysis, the (COI) genes vary significantly between *A*. *cantonensis* and *A*. *malaysiensis* (p distance = 11.1%-11.7%).

This study confirmed that the *A*. *cantonensis/mackerrasae* complex is prevalent [3.00% ± 0.76% (CI 95%)] in the molluscs surveyed from several suburbs in the Sydney metropolitan region (Table 1). Parasites were detected in two species of mollusc, *C*. *aspersum* and *B*. *similaris* which are ubiquitous in the Sydney region. To our knowledge, this is the first study to confirm that *C*. *aspersum* is a natural reservoir for *A*. *cantonensis*. This corroborates the findings of Yong *et al*. [28] who stated that *C*. *aspersum* could be infected with *A*. *cantonensis* experimentally, in contrast to an earlier study which suggested incorrectly that *C*. *aspersum* was an impractical host [29]. Admittedly, the parasite burdens calculated in this study were comparatively low (approximately 20–40 larvae in each specimen). Studies carried out elsewhere have reported parasitic burdens in excess of 1000 parasites with an estimated of 2.8 million larvae in one case involving an infected *Laevicaulis alte* [10].

*Angiostrongylus cantonensis* is known to infect species in more than 34 mollusc families, though some species of molluscs are more efficient hosts than others. Some of these common natural hosts of *A*. *cantonensis* (e.g. *Achatina fulica*, *Parmarion martensi* and *Pomacea canaliculata*) have not been reported in Australia, to our knowledge. However, some notable natural hosts for *A*. *cantonensis* are found in Sydney and mostly include several species of Limacidae (a family which includes certain species of terrestrial slug). The Limacidae includes the leopard slug; *Limax maximus*, which can reportedly harbour over 1000 parasites within 5mg of tissue [10]. *Limax maximus* was thought to be responsible for the transmission of *Angiostrongylus* to humans in the clinical cases of angiostrongyliasis reported in Sydney [22–24]. However, this study did not detect the *A*. *cantonensis/mackerrasae* complex in any of the Limacidae spp. surveyed.

Using experimentally infected molluscs (*Austropeplea lessoni*), this study confirmed that the mantle and foot of molluscs are common sites of infection for *Angiostrongylus cantonensis*. This is in agreement with previous work [15]. For larger mollusc specimens, for which it was not feasible to extract DNA from the entire specimen, DNA was extracted from mantle and foot only. Using the same experimentally infected *A*. *lessoni*, this study also found that *A*. *cantonensis* L_3_ larvae are indeed shed in mollusc mucus, although compared to thousands of L3 *A*. *cantonensis* in the snails only small numbers were found in the mucus, as reported in previous studies [10]. The finding of live *A*. *cantonensis* larvae in mollusc mucus suggests slime trails as a potential source of infection. Despite this, the infectious dose required to establish a clinically apparent *A*. *cantonensis* infection is unknown [8]. It is possible that L_3_ larvae from mollusc mucus left on raw vegetables may not provide a sufficient dose to establish an infection.

This study indicates that the *A*. *cantonensis/mackerrasae* complex is prevalent in the Sydney area. The presence of both these metastrongyloid nematodes in Australia has been known since 1968 [2], though this is the first study to use molecular testing to establish the prevalence of these parasites in molluscs in the Sydney region. We also report that *A*. *cantonensis* and *A*. *mackerrasae* are almost identical at two genetic loci (the ITS1 and 18S rRNA genes) which may indicate that these are the same species (or subspecies). However, this requires support by further molecular testing, and possibly mating studies to determine whether an *A*. *cantonensis*/*A*. *mackerrasae* cross is capable of producing fertile offspring. This study confirms that *C*. *aspersum* (and possibly *B*. *similaris*) is a natural host of the *A*. *cantonensis/mackerrasae* complex in the greater Sydney region. These molluscs are ubiquitous in the area, which probably affords these nematodes ample opportunities to come into contact with humans, and the dogs they share their environment with. Therefore, it is recommended that angiostrongyliasis be considered in all cases of eosinophilic meningitis encountered in this jurisdiction.

## Supporting Information

S1 Table Complete list of primers used within this study. (PDF)
