# Supplementary material for: The Prevalence of Angiostrongylus cantonensis/mackerrasae Complex in Molluscs from the Sydney Region
Source: PLoS One. 2015 May 22;10(5):e0128128. doi: 10.1371/journal.pone.0128128 (PMC4441457; doi:10.1371/journal.pone.0128128)
Supplement: S1 Table — (PDF) [file pone.0128128.s001.pdf]

Table S1: Complete list of primers used within this study

Primer Name	Target	Sequence (5’ – 3’)	Reference
AcanITS1F1	ITS1	Forward- 5’-TTCATGGATGGCGAACTGATAG-3’	Qvarnstrom et al. [16]
AcanITS1R1	ITS1	Reverse - 5’ –GCGCCCATTGAAACATTATACTT-3’	Qvarnstrom et al. [16]
AcanITS1P1	ITS1	Probe-5’ -6-carboxyfluroescein-ATCGCATATCTACTATACGCATGTGACACCTG-BHQ-3’	Qvarnstrom et al. [16]
AngioF1674	ITS1	Forward – 5’GTCGTAACAAGGTATCTGTAGGTG-3’	Qvarnstrom et al. [16]
58SR4	ITS1	Reverse - 5’ –TAGCTGCGTTTTTCATCGATA-3’	Qvarnstrom et al. [16]
AngioF1	18S rRNA	Forward – 5’-ATCATAAACCTTTTTTCGAGTATCCAG-3’	Qvarnstrom et al. [15]
AngioR1	18S rRNA	Reverse – 5’-TCTCGAGACAGCTCAGTCCCGG-3’	Qvarnstrom et al. [15]
NEM2F	18S rRNA	Forward- 5’-GCGGTTAAAAAGCTCGTAGTTGG-3’	Qvarnstrom et al. [15]
NEM2R	18S rRNA	Reverse-5’-CCAACTACGAGCTTTTTAACCGC-3’	Qvarnstrom et al. [15]
NEM3F	18S rRNA	Forward- 5’- GCGGCTTAATTTGACTCAACAC-3’	Qvarnstrom et al. [15]
NEM3R	18s rRNA	Reverse-5’-GTGTTGAGTCAAATTAAGCCG-3’	Qvarnstrom et al. [15]
F-573	18S RNA	Forward- 5’-CGCGGTAATTCCAGCTCCA- 3’	Hadziavdic et al. [26]
R-1200	18S RNA	Reverse- 5’- CCCGTGTTGAGTCAAATTAAGC-3’	Hadziavdic et al. [26]
